# Supplementary material for: Cellular and Molecular Targets of Nucleotide-Tagged Trithiolato-Bridged Arene Ruthenium Complexes in the Protozoan Parasites Toxoplasma gondii and Trypanosoma brucei
Source: Int J Mol Sci. 2021 Oct 5;22(19):10787. doi: 10.3390/ijms221910787 (PMC8509533; doi:10.3390/ijms221910787)

Toxoplasma gondii **OD62 binding proteins interaction mapping by STRING**

Network statistics

number of nodes: 60
number of edges: 106
average node degree: 3.53
avg. local clustering coeff.: 0.394
expected number of edges: 75
PPI enrichment p-value: 0.000459

Translation

Proteasome

Myosine

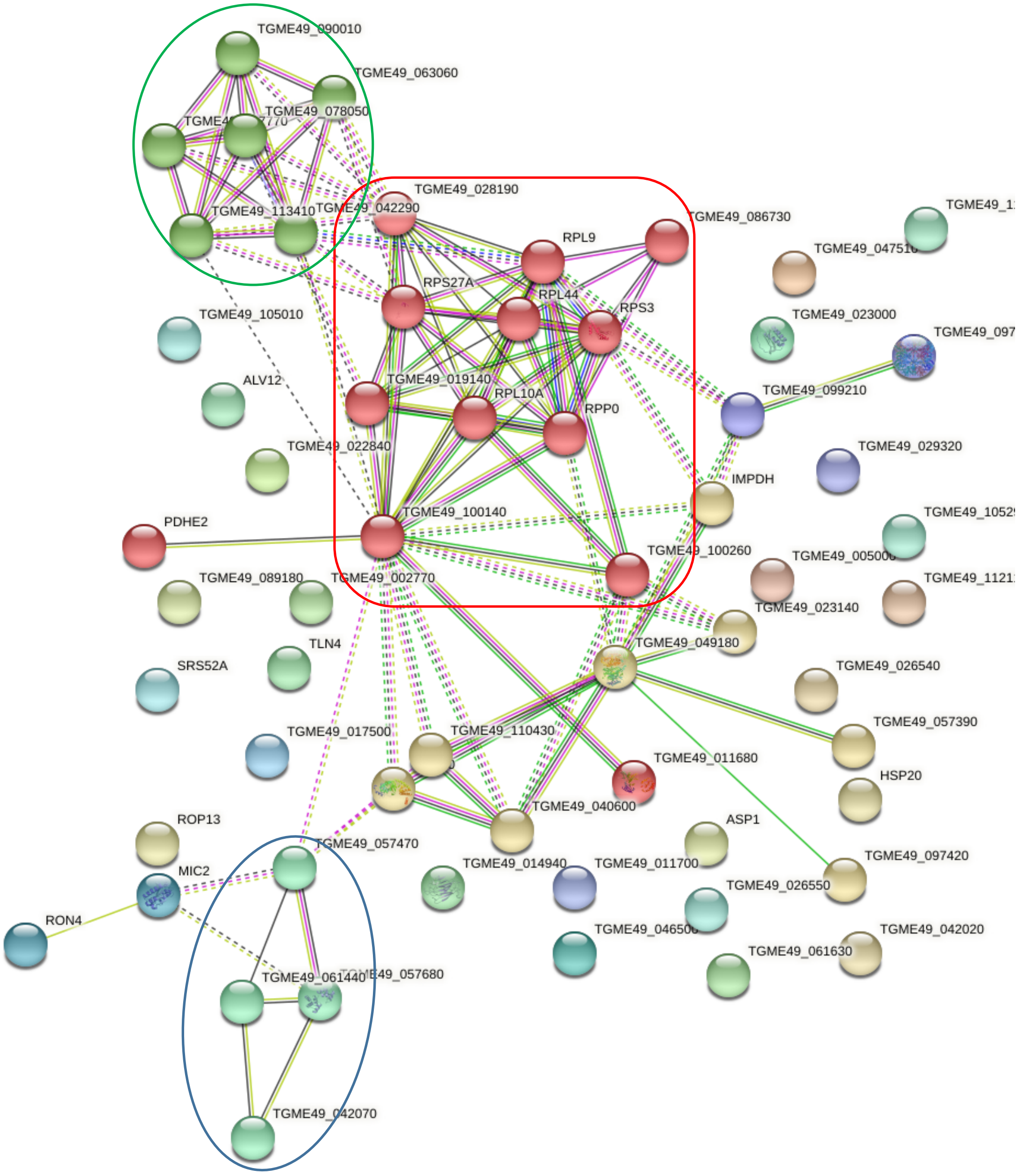

Supplement: Supplementary file 1 [file ijms-22-10787-s001.zip › Figure S1.pdf]
