# Supplementary material for: Cellular and Molecular Targets of Nucleotide-Tagged Trithiolato-Bridged Arene Ruthenium Complexes in the Protozoan Parasites Toxoplasma gondii and Trypanosoma brucei
Source: Int J Mol Sci. 2021 Oct 5;22(19):10787. doi: 10.3390/ijms221910787 (PMC8509533; doi:10.3390/ijms221910787)

Trypanosoma brucei **OD62 binding proteins interaction mapping by STRING**

Network statistics

number of nodes:	22
number of edges:	17
average node degree:	1.55
avg. local clustering coeff.:	0.483
expected number of edges:	10
PPI enrichment p-value:	0.0315

ATP synthase

Translation

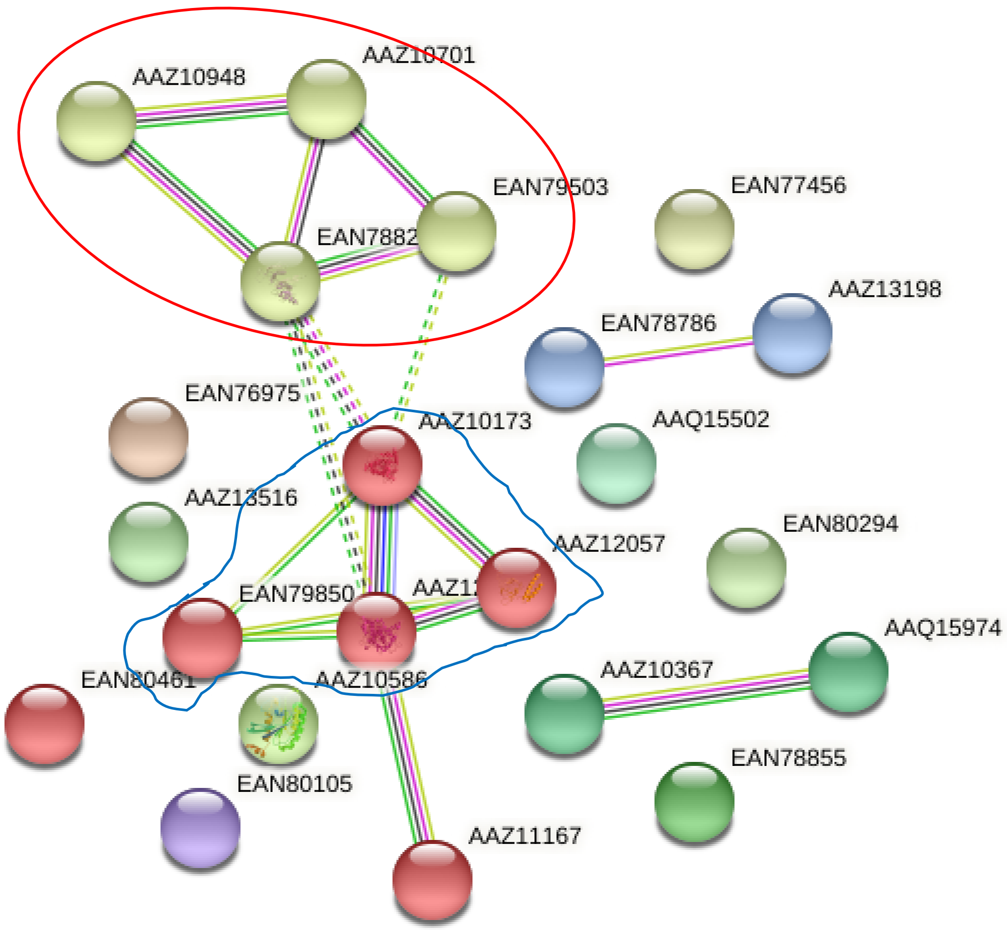

Supplement: Supplementary file 1 [file ijms-22-10787-s001.zip › Figure S2.pdf]
